# Editorial: Stress erythropoiesis

**DOI:** 10.3389/fphys.2023.1165315

**Published:** 2023-02-22

**Authors:** Jaira Ferreira de Vasconcellos, Emily Riehm Meier, Nermi Parrow

**Affiliations:** ^1^ Department of Biology, James Madison University, Harrisonburg, VA, United States; ^2^ Global Blood Therapeutics, South San Francisco, CA, United States; ^3^ Department of Pediatrics, Saint Louis University School of Medicine, St Louis, MO, United States

**Keywords:** ineffective erythropoiesis, erythropoiesis, hematopoiesis, anemia, iron metabolism

Stress erythropoiesis broadly refers to the extramedullary production of red cells in response to influences that result in inadequate marrow production. A variety of transcription factors have been implicated in the regulation of the stress erythropoiesis response. Examples include p53 *via* ROCK1 (Vemula et al., 2012), Wnt (Chen et al., 2020), glucocorticoids (Flygare et al., 2010), and members of the Transforming Growth Factor beta (TGF-β) superfamily, among others (Perry et al., 2007; Hao et al., 2019). Whether the myriad of regulators are involved in distinct pathways or converge on a single pathway is not entirely clear. Elucidation of the molecular mechanisms of stress erythropoiesis has important implications for several conditions and disease states. These include iron deficiency anemia, anemia of inflammation, cancer related anemia, hemoglobinopathies, hemorrhage, injury and others. The contributions to this Research Topic are focused on understanding of the molecular mechanisms of stress erythropoiesis. Experimental models used to study stress erythropoiesis and clinical trials of therapeutic approaches are also highlighted.

The role of iron in the regulation of erythropoiesis is explored in two original research articles. Using mouse models of dietary iron deficiency with or without anemia, Li et al. investigated the effects of exogenous iron-loaded transferrin on erythropoiesis and iron metabolism. Of importance, administration of exogenous iron-loaded transferrin restored serum iron and transferrin saturation levels in mice with iron deficiency without anemia to levels similar to control mice. Transferrin administration also increased hepcidin levels without increasing the iron regulatory molecule BMP6 in these mice. In mice with iron deficiency anemia, administration of iron-loaded transferrin did not further increase serum erythropoietin but did increase the expression level of the erythropoietin target gene, erythroferrone (*Erfe*). These observations suggest that iron loaded transferrin influences hepcidin expression and erythropoietin sensitivity, in accordance with the previously described role of transferrin as a signaling molecule (Parrow et al., 2019).

Using an iron-loaded thalassemia intermedia mouse model (Hbb th3/+; Yang et al., 1995). investigated the effects of green tea extract (GTE) on erythropoiesis. GTE has antioxidant and iron-chelating properties and can inhibit non-heme iron absorption (Samman et al., 2001). Suppression of the iron regulatory hormone hepcidin by excessive erythropoietic signaling is thought to contribute to the pathophysiology of beta thalassemia. Accordingly, decreasing iron absorption by increasing hepcidin has previously been shown to ameliorate disease in the Hbb th3/+ mouse model (Gardenghi et al., 2010; Guo et al., 2013). GTE treatment was hypothesized to have an effect on erythroid regulators and iron mobilization in these mice. Interestingly, iron-loaded thalassemia mice treated with GTE made fewer red cells resulting in a net decrease in hematocrit, despite relatively higher mean cellular volume and mean cellular hemoglobin, compared to untreated iron-loaded thalassemic mice and those treated with the iron chelator deferiprone (DFP) or DFP combined with GTE. Nonetheless, GTE treated mice demonstrated similar reductions in the levels of plasma erythropoietin and the erythroid factor erythroferrone (Erfe) as mice treated with DFP or DFP combined with GTE. Expression levels of splenic *Erfe* and renal *Epo* followed a similar pattern. All treatments likewise decreased tissue iron concentrations as previously reported (Koonyosying et al., 2018). Overall, this study suggests that treatment with GTE ameliorates iron dysregulation in iron-overloaded *β*-thalassemic mice in a manner distinct from that of iron chelation *per se*. Its precise mechanism of action and how it influences iron availability to the erythron would be interesting areas of future studies.

Stress erythropoiesis was reviewed from different perspectives in the remaining contributions. The basic cellular metabolism of stress erythroid progenitors was explored by Ruan and Paulson. They reviewed the alterations in cellular metabolism that accompany the expansion stage of stress erythropoiesis and delineated a series of proposed metabolic adaptations that are likely to drive erythroid proliferation based on pathways described in other cell types. Future investigations of these proposed changes in cellular metabolism are likely to be especially interesting as they may identify novel therapeutic targets to regulate stress erythropoiesis.

In a more clinical vein, Petrinović et al. reviewed stress erythropoiesis during tumor progression. Cancer related anemia is a common condition that can arise from the cancer itself, as well as from chemotherapies, infections and other comorbidities. Each of these factors may contribute to anemia by decreasing production or increasing destruction of red cells, or by causing blood loss (Gilreath and Rodgers, 2020). The review focused on molecular pathways and signaling molecules known to modulate cancer-related anemia, such as the erythropoietin/erythropoietin receptor axis and select TGF-β signaling pathways, emphasizing the potential for stress erythropoiesis to promote conditions that favor ineffective erythropoiesis.

In another clinically relevant topic, Munley et al. reviewed post-traumatic erythropoiesis and persistent anemia, a state that can persist for up to 6 months after injury. This article focused on known mediators of persistent anemia, such as prolonged hypercatecholaminemia after injury. Importantly, the article also focused on evidence supporting strategies for adrenergic modulation of erythropoiesis after trauma, including alpha-2 agonism and selective or non-selective blocking of beta-1, beta-2 or beta-3 receptors. The authors propose several areas of future investigation, including clinical studies of therapeutics with promising preclinical findings and assessment of potential effects of age and type of trauma on post-traumatic management of persistent anemia.

Collectively, the contributions to this Research Topic provide insights into the role of iron metabolism in the regulation of stress erythropoiesis and a sampling of important clinical conditions characterized by stress erythropoiesis. They also highlight the need for a deeper understanding of the basic biology of stress erythropoiesis, one that can be translated to effective therapeutic approaches for the treatment of anemia in these conditions and others. Continued investigations of the nature of the stress erythroid progenitor(s) and signals they respond to are needed within the discipline. We look forward to the exciting results that are likely to be reported in the future studies of stress erythropoiesis proposed by the contributors of this Research Topic.
